# Estimation of Pubertal Growth-Spurt Parameters in Children and Adolescents in Colombia: Comparison between Low and Moderate Altitudes

**DOI:** 10.3390/jcm11133847

**Published:** 2022-07-02

**Authors:** María Correa-Rodríguez, Rossana Gomez-Campos, Marco Antonio Cossio-Bolaños, Florelba Campo-Lucumí, Katherine González-Ruíz, Robinson Ramírez-Vélez

**Affiliations:** 1 Nursing Department, Faculty of Health Sciences, University of Granada, 18010 Granada, Spain; macoro@ugr.es; 2 Institute of Biomedical Research (IBS), 18012 Granada, Spain; 3 Facultad de Ciencias de la Educación, Programa de Doctorado en Ciencias de la Actividad Física, Universidad Católica del Maule, Talca 3466706, Chile; rossaunicamp@gmail.com (R.G.-C.); mcossio30@hotmail.com (M.A.C.-B.); 4 Grupo de Investigación en Estudios Aplicados al Deporte, Institución Universitaria Escuela Nacional del Deporte, Cali 760042, Colombia; lucumiflor@gmail.com; 5 Grupo de Investigación Salud y Movimiento, Programa de Fisioterapia, Facultad de Salud, Universidad Santiago de Cali, Cali 760035, Colombia; katherine.gonzalez07@usc.edu.co; 6 Navarrabiomed, Hospital Universitario de Navarra (HUN), Universidad Pública de Navarra (UPNA), IdiSNA, 31006 Pamplona, Spain; 7 CIBER of Frailty and Healthy Aging (CIBERFES), Instituto de Salud Carlos III, 28029 Madrid, Spain; 8 Facultad de Ciencias de la Educación, Unidad Central del Valle del Cauca (UCEVA), Tuluá 763022, Colombia

**Keywords:** growth velocity, Preece–Baines method, altitude, children, adolescents

## Abstract

Context-specific information, including differences in geographical areas, such as distinct altitudes, can be important to explain variations in physical growth. We aimed to compare the estimation of maximum growth velocity and pubertal growth-spurt parameters of children and adolescents living at low and moderate altitudes in Colombia. A cross-sectional study, including a representative cohort of 30.305 (51% boys) children and adolescents aged 1–18 years from Colombia, was performed. The heights were measured with standardized techniques. The Preece–Baines growth model was used to estimate the mathematical and biological parameters of the height-growth velocities and growth spurts for both sexes. The altitudes were categorized as low (18 to 564 m above sea level) or moderate (2420 to 2640 m above sea level). There were no differences in final height (*h*_1_), peak height velocity size (*h_θ_*), age at peak height velocity (APHV), or peak height velocity PHV (cm/y) between the subjects living in both altitudes (*p* > 0.05). The APHV was estimated at 12.75 ± 0.75 years in the boys and at 10.05 ± 0.65 years in the girls. The girls reached the APVH 2.70 years earlier than the boys. Regarding the PHV, the boys reached higher growth velocity, which was 6.85 ± 0.55 cm/y. In conclusion, there were no significant differences in final height, peak height, APHV, or PHV between the children and adolescents living at distinct altitudes in Colombia. The PHV occurred approximately 3 years earlier in the girls than in the boys. Furthermore, the girls’ estimated PHV, APHV, and final height were lower than those of the boys. This study allows additional insight into pubertal growth-spurt parameters and also provides a valuable reference database for the assessment of Colombian children and adolescents.

## 1. Introduction

Physical growth is a complex process, resulting from hyperplasia, hypertrophy, and cellular aggregation that determines progressive changes in body dimensions [1]. It is characterized by its extraordinary plasticity and population heterogeneity [2]. General growth varies according to chronological age in childhood and adolescence; girls often have earlier growth and reach their final size earlier than boys of the same age [3,4]. The pattern of pubertal growth varies according to the age of the onset of the pubertal growth spurt, which occurs within a 5-year period (e.g., in females aged 8 to 13 years and in males aged 10 to 15 years) [5,6]. During this period, girls average a maximum height velocity of 9 cm/year at age 12, and boys, on average, reach a maximum height velocity of 10.3 cm/year at age 14 [7].

The variability of growth velocities can be investigated by constructing growth-velocity curves and estimating the mathematical and biological parameters, using mathematical models, which allows the gathering of information on timing and tempo [8]. In this context, the age of the peak height velocity (APHV) (years) and the peak height velocity (PHV) (cm/years) are the non-invasive biological parameters that can be estimated through the mathematical model of Preece and Baines [9]. This model belongs to a family of curves that fit the shape of the human growth curve. In fact, several recent studies have focused on modeling linear physical growth with cross-sectional data from children and adolescents living in various regions of the world [3,5,10]. 

It has been proposed that context-specific information, including differences in geographical area, such as distinct altitudes, can be important to explain variations in physical growth [10,11,12,13,14,15]. These differences are apparent in Colombia, whose territory spreads across areas with different altitudes. Interestingly, impaired growth among children living at high-altitude sites in the United States was demonstrated by the study by Yip et al., conducted on children aged 5 years or younger [11]. Furthermore, a previous large-scale study reported that children living 1500 m or more above sea level follow a lower growth trajectory than their peers living at lower altitudes [12]. 

In Colombia, Cossio-Bolaños estimated the final height, age at peak height velocity (APHV), and peak height velocity PHV (cm/y) of children and adolescents living at moderate altitudes [16]. Furthermore, a recent review examined the heights of children and teenagers in Colombia based on studies published since 1957, showing that the anthropometric indicators in the country have changed over time [17]. Previous studies conducted on children and adolescents living at different altitudes reported inconsistent results. Thus, Santos et al. recently showed that physical-growth timing and tempo are influenced by the altitudes at which people live [10] whereas Freyre and Ortiz revealed relatively little effect of altitude upon growth or final attained height [18]. However, although it is of significant interest to understand population differences in terms of growth, there is no available information regarding the comparison of growth velocity curves and pubertal spurt parameters in Colombian children and adolescents living at low and moderate altitudes. The provision of reference values for growth and development is important, since the growth assessment of children and adolescents requires a comparison of growth measurements with normative references. Growth curves by age and sex have important implications for the control of child-health programs and clinical and epidemiological practices. Based on previous research, in this study, we hypothesized that altitude could have an impact on pubertal growth-spurt parameters of children and adolescents in Colombia. 

In this context, this study aimed to compare, for the first time, the estimation of the maximum growth velocity and pubertal growth-spurt parameters of children and adolescents living at low and moderate altitudes in Colombia.

## 2. Materials and Methods

This was a cross-sectional study using representative data collected in 2015–2016 by the National Nutritional Situation Survey in Colombia (in Spanish, Encuesta Nacional de Situación Nutricional ENSIN-2015). Data from ENSIN-2015 are anonymized, and made public; they can be accessed through a well-founded request to the Colombian Ministry of Health. The ENSIN-2015 was carried out over the past 4 years in Colombia. It was designed to represent 99% of the population through stratified multistage sampling. Subjects were selected with various sampling methods, including probability, cluster, stratified, and multistage sampling. A total of 44,202 households were surveyed, representing 4739 groups of 295 strata (*n* = 151,343 people, from 1 to 17 years, adults aged between 18 and 64 years, and pregnant girls and women). All selected households were included in the demographic and anthropometric components. The subpopulation analyzed included 36,366 (51% boys) children and adolescents aged 1–18 years. 

**Figure 1:**
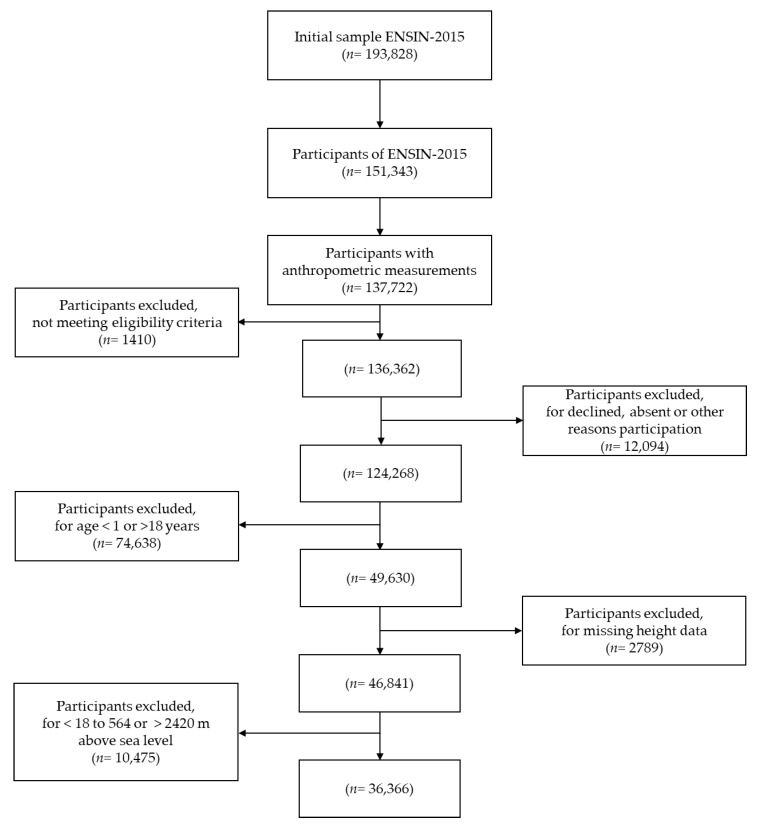
Flowchart depicting final study-subject selection from the ENSIN-2015 population.

Data were collected by trained nutritionists in household settings between December 2015 and November 2016, using computer-assisted personal-interview technology, which can detect inconsistencies. All data-collection procedures and performances of the interviewers were tested in a pilot study and standardized through procedure manuals. The ENSIN protocol was approved by the Profamilia Institutional Review Board on Research involving Human Subjects and the Colombian National Institutes of Health (file number 2-2015, 26 February 2015). The methodological details were published previously [19], and Figure 1 shows application of inclusion and exclusion criteria.

Standing height without shoes was measured in the Frankfort horizontal plane (the plane joining the lower border of the left orbit and the upper margin of the external auditory meatus in a horizontal position) to the nearest 0.1 mm using a stadiometer (Diseños Flores S.R., Ltda., Bogotá, Colombia). Body weight was established with SECA scales (Model 874^®^ Hamburg, Germany). Body mass was measured with participant standing without shoes in light indoor clothes. 

To infer the mathematical and biological parameters of height-growth velocity and growth spurt for both sexes, the 1PB [9] nonlinear regression model 1 was used. Thereafter, children and adolescents were classified as low (18 to 564 m above sea level) or moderate (2420 to 2640 m above sea level) according to altitude. The low-altitude group represented the sub-regions and cities of Barranquilla, Atlántico, San Andrés, Bolívar, Sucre, Córdoba, Cali, Guajira, Cesar, Magdalena, Litoral Pacifico, Orinoquia and Amazonia, Tolima, Huila, Caquetá, and Valle, and moderate-altitude group represented Bogotá, Boyacá, and Cundinamarca sub-region.

The Kolmogorov–Smirnov test was used to evaluate data-distribution normality. Descriptive statistics (mean, standard deviation, range) were calculated. Welch’s *t*-test was used to compare the two groups of low and high altitude (it is an unbiased test for similar or different variances and is usually applied to compare all sample sizes). The independent two-sample *t*-test was used to analyze differences between both sexes. The 1PB model was used to estimate the mathematical and biological parameters. Five parameters were calculated according to the following equation:

(1) $$h=h_{1}-\frac{2\left(h_{1}-h_{\theta }\right)}{e^{s_{0\left(t-\theta \right)}}+e^{s_{1\left(t-\theta \right)}}}$$

Here, *h*_1_—final height (cm), *h_θ_* and *θ*—average height (cm) and age (years) for height on the decreasing slope of the PHV, *s*_0_ and *s*_1_—prepubertal and pubertal rate constants controlling growth rate (cm/y). We also determined the effect size of the difference between the two groups using Cohen’s *d* test. The effect size was considered small (Cohen’s *d* = 0.2), medium (Cohen’s *d* = 0.5), or large (Cohen’s *d* = 0.8). The calculations and graphs of the curves were obtained by means of the computer program implemented in the software R version 4.1.3 (R Core Team, 2020, China). Statistical significance was set at *p* value less than 0.05.

## 3. Results

The mean and standard deviation values of the heights of the children and adolescents according to age, sex, and altitude group are shown in Table 1. According to Welch’s test, the results indicate that there were no significant differences in height between the low- and moderate-altitude groups in any age or sex group (*p* > 0.05). In addition, the confidence intervals (CI: 95%) of the difference between the means crossed zero at all ages and in both sexes, demonstrating the similarity in the heights between the two groups, despite the fact that the low-altitude sample size was larger than the high-altitude sample size. Cohen’s *d* also indicated small effect sizes between the low and high altitudes.

The mathematical and biological parameters of the statures of the children and adolescents according to sex and altitude group are presented in Table 2. The five mathematical parameters estimated by model 1 PB demonstrate small residuals at both low and moderate altitudes (in boys 0.39 and 0.38, and in girls 0.36 and 0.35). There were no differences in final height (*h*_1_) and peak height velocity size (*h_θ_*) between both altitude levels, in both boys and girls. Regarding the biological parameters, the APHV and the PHV were also similar at both altitudes and in both sexes. 

**Figure 2:**
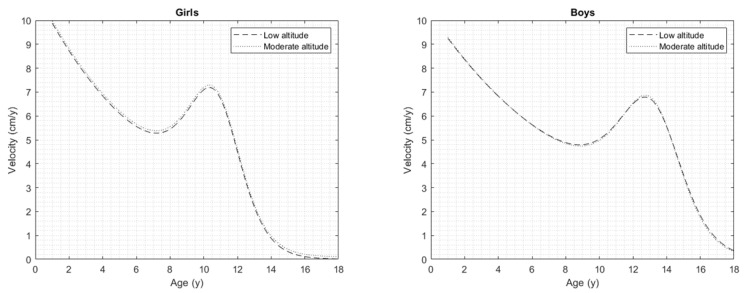
Comparison of the PHV curves of children and adolescents of both sexes living at low and moderate altitudes in Colombia.

Figure 2 shows a comparison of the PHV curves of the children and adolescents of both sexes living at low and moderate altitudes in Colombia.

**Figure 3:**
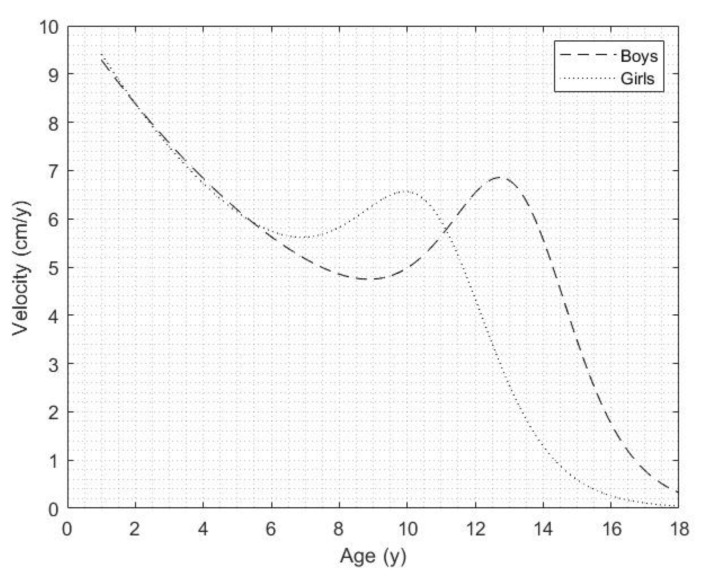
Peak height velocity curves of Colombian children and adolescents living at both altitudes by sex.

Figure 3 shows a comparison of the PHV curves of the Colombian children and adolescents living at both altitudes by sex. The APHV was estimated at 12.75 ± 0.75 years in the boys and at 10.05 ± 0.65 years in the girls. Thus, the girls reached the APVH 2.70 years earlier than the boys. Regarding the PHV, the boys reached higher growth velocity in terms of height, which was 6.85 ± 0.55 cm/y whereas in the girls, it was 6.55 ± 0.54 cm/y.

## 4. Discussion

In this study, we investigated the estimation of maximum growth velocity and pubertal growth-spurt parameters of a large cohort of 30,305 children and adolescents living at low and moderate altitudes in Colombia. We observed that there were no significant differences in the mathematical parameters, including the final height and peak height velocity size, or in the biological parameters, such as the APHV and the PHV, between the subjects living at both altitudes in relation to sex or age, suggesting that altitude does not play a relevant role in pubertal growth-spurt parameters. In addition, using a nationally representative sample of Colombian children and adolescents, this study provided the PHV curves of subjects living at distinct altitudes by sex. The presented curves by age and sex could have important implications for child-health programs and in clinical and epidemiological practices.

In line with our results, a previous study assessing the effect of altitude on adolescent growth and development in subjects who resided at sea level (*n* = 1262 subjects), mid-altitude (*n* = 1743 subjects), and high altitude (*n* = 1137 subjects) revealed relatively little effect of altitude upon growth and final attained heights [18]. By contrast, Santos et al. reported that physical growth timing and tempo were influenced by the altitudes at which a cohort of 10,795 Peruvian children and adolescents lived [10]. Those living at sea level (58 m) experienced PHV at an earlier age, were taller at the time of their PHV, had a higher PHV, and had a taller estimated final height compared to those living at higher altitudes (751 m and 4107 m). Similarly, highland infants were reported to have a higher prevalence of reduced height in Jujenean (Argentina) infants aged 1–4 years (highlands ≥ 2500 masl and lowlands < 2500 masl) [20]. It has been proposed that the differences in children’s height are likely not to be due to genetic differences between subjects. They might be caused by environmental factors, including the continual exposure of children to poverty, a lack of maternal education, and a lack of access to safe water and sanitation across populations [21]. Furthermore, a recent study investigating whether altitude is associated with increased risk of linear growth faltering in children aged 0 to 59 months in the range from sea level up to 1700 m (Austria) suggests that residing at a higher altitude may be associated with the slowing of child growth [12]. However, the authors indicated that the association between altitude and growth is more pronounced during the perinatal period. Our study was carried out on a cohort of subjects aged 1–18 years, which differed from others and, therefore, might explain the inconsistent results. 

The results of this study demonstrated that geographical differences regarding altitude may not lead to differences in pubertal growth-spurt parameters in Colombian children and adolescents, suggesting that high-altitude residence per se might not be a risk factor for growth faltering. In fact, the heights of children and adolescents living at low and moderate altitudes showed a small effect size at each of the ages (from the first year of life to 18 years). These results suggest that residence at moderate altitude per se may not be a risk factor for growth retardation. The growth deficits observed in other studies may be manifestations of poorer living conditions, which may be more pronounced at high altitudes. Thus, the observed growth deficits might have been due to harsher living conditions and poorer diets at higher altitudes [21]. Colombia has experienced important nutritional and epidemiological transitions. Thus, the lack of variations between regions could be explained by the reduction in the disparities between different populations, the improvement of living conditions in Colombia, and lifestyles, including food habits and physical activity levels. 

The findings of this study show that in children and adolescents living at low and moderate altitudes in Colombia, the APHV was reached at 12.75 ± 0.75 years in the boys and at 10.05 ± 0.65 years in the girls, indicating that the girls reached the APVH approximately 3 years earlier than the boys. Girls experience their mid-growth spurt and their adolescent spurt at earlier ages and undergo a more rapid pubertal transition, whereas boys have a substantially longer growth period [8]. The reported values are relatively similar to those presented by Cossio-Bolaños et al. in a recent study conducted on a Colombian population [16]. They reported that the APHV was estimated at 12.708 ± 0.131 and 10.422 ± 0.195 years in boys and girls, respectively. Similarly, the authors indicated that girls reached their APHV 2.23 years earlier than boys. Nevertheless, the values identified in our study show earlier ages of APHV compared to a study conducted on a sample of 1039 schoolchildren from el Yopal (Colombia) [22]. Ireton et al. reported that the APHV was reached at 13.6 years in boys and 11.1 years in girls.

Regarding the PHV, this study demonstrates that the boys reached a higher growth velocity, which was 6.85 ± 0.55 cm/y, whereas, in the girls, it was 6.55 ± 0.54 cm/y, suggesting that Colombian girls and boys living at both altitudes differed in their PHV. These velocity patterns were less accelerated than similar patterns from children and adolescents living at moderate altitudes in Colombia (7.43 ± 0.42 cm/y in boys and 7.00 ± 0.19 cm/y in girls) [16]. However, our data were similar to those reported from Yopal (Colombia), which demonstrated 6.96 years in boys and 6.57 years in girls [22].

The available data regarding the physical growth and pubertal growth-spurt parameters of children and adolescents from different regions of Colombia are scarce. To the best of our knowledge, this is the first study to analyze these data among a nationally representative sample of Latin American subjects living at low and moderate altitudes. We have contributed to the literature by using a larger cohort of Colombian children and adolescents, and the novel information provided in this study may be of special interest to professionals working in clinical and epidemiological contexts, allowing the comparisons of pubertal growth-spurt parameters between different populations.

This study has some limitations that should be mentioned. Firstly, it should be highlighted that since this was a cross-sectional study, the 1PB model could underestimate the biological parameters, mainly in girls [23]. Furthermore, the study’s cross-sectional nature does not allow a dynamic analysis of intraindividual changes or interindividual differences in pubertal growth-spurt parameters during childhood and adolescence. Therefore, these data should be investigated in future longitudinal studies. Secondly, the fact that our study comprised a cohort of Colombian subjects may limit the generalizability of the results to other populations. We did not link the altitudes at which the subjects lived with physiology or nutrition habits, parameters that may affect growth. Furthermore, although the analysis of the link between living altitudes and nutrition habits and the confirmation of the data in comparison to final parental height would be of interest, these data were not presented in this subpopulation. However, the strengths of this study include its nationally representative sample of Colombian children and adolescents by age and sex and its estimation, for the first time, of the maximum growth velocity and pubertal growth-spurt parameters of subjects living at low and moderate altitudes.

## 5. Conclusions

In conclusion, using a nationally representative sample of children and adolescents, we reported that there were no significant differences in final height, peak height, APHV, or PHV between subjects living at distinct altitudes. The PHV occurred approximately 3 years earlier in the girls than in the boys. Furthermore, the girls’ estimated PHV, APHV, and final height were lower than those of the boys. This study allows additional insight into pubertal growth-spurt parameters and also provides a valuable reference database for the assessment of Colombian children and adolescents. 

## Figures and Tables

**Table 1:** Descriptive values (mean ± SD) of the heights of Colombian children and adolescents according to age, sex, and altitude groups.

Age (Years)	Girls									Boys								
		Low			Moderate		*p*	95% CI	Cohen’s *d*		Low			Moderate		*p*	95% CI	Cohen’s *d*
		Altitude			Altitude						Altitude			Altitude				
	*n*	Mean	SD	*n*	Mean	SD				*n*	Mean	SD	*n*	Mean	SD			
1	799	78.50	5.73	152	78.44	5.32	0.9048	−1.044 to 0.924	0.010	810	79.94	5.06	168	79.52	7.99	0.3824	−1.363 to 0.523	0.060
2	833	87.15	5.15	164	86.83	4.66	0.4605	−1.170 to 0.531	0.065	836	88.06	4.89	172	88.62	7.44	0.2165	−0.328 to 1.449	0.088
3	810	94.98	4.80	163	95.50	5.16	0.2131	−0.299 to 1.339	0.104	828	96.15	5.37	147	96.11	4.19	0.9317	−0.955 to 0.875	0.008
4	843	102.30	5.75	163	102.82	6.25	0.2996	−0.463 to 1.503	0.080	851	102.95	5.38	182	102.38	5.29	0.1939	−1.430 to 0.290	0.106
5	806	108.49	6.71	136	108.57	5.05	0.8944	−1.102 to 1.262	0.010	849	109.56	5.72	166	109.12	9.11	0.4178	−1.505 to 0.625	0.057
6	842	114.61	7.14	169	114.57	5.18	0.9448	−1.173 to 1.093	0.006	891	115.79	6.18	175	114.85	5.94	0.0644	−1.936 to 0.056	0.155
7	915	120.45	6.45	160	119.92	5.62	0.3290	−1.595 to 0.535	0.087	898	121.26	6.44	186	120.74	5.43	0.3042	−1.513 to 0.472	0.087
8	850	126.06	7.76	196	125.85	6.57	0.7257	−1.384 to 0.964	0.029	973	126.66	6.62	172	126.63	5.28	0.9551	−1.075 to 1.015	0.005
9	849	132.18	7.52	167	131.42	5.80	0.2170	−1.967 to 0.447	0.110	964	130.87	6.63	195	131.60	7.02	0.0319	−0.301 to 1.761	0.160
10	830	138.31	9.60	151	138.10	7.20	0.7980	−1.820 to 1.400	0.027	902	136.14	7.35	180	136.11	6.55	0.9594	−1.187 to 1.127	0.004
11	903	144.70	7.99	158	144.77	7.81	0.9188	−1.278 to 1.418	0.000	955	141.23	8.12	184	140.67	7.35	0.3848	−1.824 to 0.703	0.072
12	861	150.30	7.06	162	150.16	7.10	0.8171	−1.328 to 1.048	0.019	928	146.83	9.62	184	147.25	9.15	0.5856	−1.091 to 1.931	0.040
13	839	153.38	6.65	184	153.27	5.93	0.8360	−1.153 to 0.932	0.017	862	153.78	9.55	189	153.00	9.13	0.3057	−2.273 to 0.713	0.083
14	842	155.35	6.23	152	155.19	5.85	0.7688	−1.228 to 0.907	0.026	849	160.46	9.16	155	160.83	8.54	0.6405	−1.184 to 1.924	0.041
15	867	156.17	7.21	190	155.38	5.84	0.1582	−1.888 to 0.307	0.120	806	164.77	7.96	193	164.79	7.38	0.9746	−1.215 to 1.255	0.003
16	819	156.94	6.13	175	156.92	6.02	0.9687	−1.019 to 0.978	0.003	871	167.68	7.72	157	167.98	5.83	0.6430	−0.969 to 1.570	0.043
17	709	156.39	8.19	159	156.78	6.13	0.5716	−0.962 to 1.743	0.050	746	168.26	7.35	132	167.79	7.18	0.4970	−1.828 to 0.887	0.064
18	644	157.10	6.01	171	156.58	6.55	0.3241	−1.555 to 0.514	0.082	625	168.78	7.17	152	168.93	5.89	0.8112	−1.082 to 1.382	0.022

SD: standard deviation. 95% CI: 95% confidence interval.

**Table 2:** Mathematical and biological parameters of height, estimated by 1 Preece–Baines (1PB) model according to sex and altitude groups.

PB Model Parameters	Boys				Girls			
	Low Altitude		Moderate Altitude		Low Altitude		Moderate Altitude	
	Mean	SE	Mean	SE	Mean	SE	Mean	SE
Mathematical parameters								
*s*_0_	0.10	0.00	0.10	0.00	0.12	0.00	0.12	0.00
*s*_1_	0.93	0.03	0.91	0.05	0.87	0.02	0.93	0.05
*θ*	13.45	0.05	13.48	0.10	11.04	0.05	11.07	0.09
*h_θ_*	156.96	0.19	156.99	0.42	145.09	0.19	145.07	0.34
*h*_1_	169.39	0.22	169.57	0.49	156.98	0.14	156.62	0.25
RSE	0.39		0.38		0.36		0.35	
Biological Parameters								
APHV (y)	12.80	0.67	12.80	0.65	10.20	0.34	9.96	0.44
PHV (cm/y)	6.86	0.45	6.78	0.35	6.67	0.76	6.54	0.56

SE: standard error, RSE: residual standard error, APHV: age at peak height velocity, PHV: peak height velocity. PB: Preece and Baines.

## Data Availability

All the data analyzed during this study are publicly accessible through a reasonable request to the Ministry of Health and Social Protection of Colombia.
